# Opkomst en neergang van ziekten in Nederland: Succes en falen van de publieke gezondheidszorg in langetermijnperspectief

**DOI:** 10.1007/s12508-022-00362-x

**Published:** 2022-10-28

**Authors:** Johan P. Mackenbach

**Affiliations:** Afdeling Maatschappelijke gezondheidszorg, Erasmus MC, Rotterdam, Nederland

**Keywords:** levensverwachting, epidemiologie, geschiedenis, public health, Europa, Nederland, Life expectancy, Epidemiology, History, Public health, Europe, Netherlands

## Abstract

De toename van de levensverwachting bij de geboorte is het resultaat van de op- en neergang van sterfte aan een groot aantal afzonderlijke ziekten. Dat zoveel ziekten een patroon van opkomst en neergang vertonen, berust op het feit dat zowel opkomst als neergang veelal een direct of indirect gevolg zijn van sociaaleconomische ontwikkelingen. Deze leiden enerzijds tot blootstelling aan nieuwe gezondheidsrisico’s, anderzijds tot meer mogelijkheden om gezondheidsrisico’s te bestrijden, in de vorm van publieke gezondheidszorg of medische zorg. Dit paradoxale verschijnsel wordt geïllustreerd aan de hand van historische Europese voorbeelden, waarbij vervolgens de vraag aan de orde komt hoe de Nederlandse ervaringen op dit vlak zich verhouden tot die van andere landen, in het bijzonder Zweden, dat al gedurende lange tijd een van de meest succesvolle landen is op het gebied van preventief gezondheidsbeleid. Alleen rond het midden van de twintigste eeuw streefde Nederland Zweden voorbij, in het bijzonder wat betreft het verlagen van de zuigelingensterfte, maar sindsdien is Nederland weer teruggezakt in een Europese ‘subtop’, onder meer door een weinig doortastend antirookbeleid. Dit wijst erop dat de publieke gezondheidszorg in Nederland beter moet kunnen door op zoek te gaan naar een succesformule die past bij de gezondheidsproblemen van de eenentwintigste eeuw.

## Abstract (en)

The increase in life expectancy at birth is the result of the rise and fall of mortality from a large number of separate diseases. The fact that so many diseases show a pattern of rise-and-fall is due to the fact that both rise and fall are often a direct or indirect consequence of socio-economic developments. These lead on the one hand to exposure to new health risks, on the other hand to more possibilities to combat health risks, in the form of public health or medical care. This paradoxical phenomenon is illustrated with historical European examples, and then the question is raised of how Dutch experiences in this area relate to those of other countries, in particular Sweden, which has long been one of the most successful countries in the field of preventive health policy. Only around the middle of the twentieth century did the Netherlands surpass Sweden, in particular in terms of reducing infant mortality, but since then the Netherlands has fallen back into a European ‘sub-top’, partly due to a lack of decisive anti-smoking policy. This indicates that public health in the Netherlands can do better, by looking for a success formula that fits the health problems of the twenty-first century.

## Inleiding

Weinig andere historische ontwikkelingen hebben ons leven zo sterk veranderd als de stijging van de levensverwachting. In Nederland kan de ontwikkeling van de levensverwachting bij de geboorte worden gevolgd vanaf de tweede helft van de negentiende eeuw, en sindsdien is de gemiddelde levensverwachting bij de geboorte gestegen van ongeveer 40 tot ruim 80 jaar.

**Figure 1:**
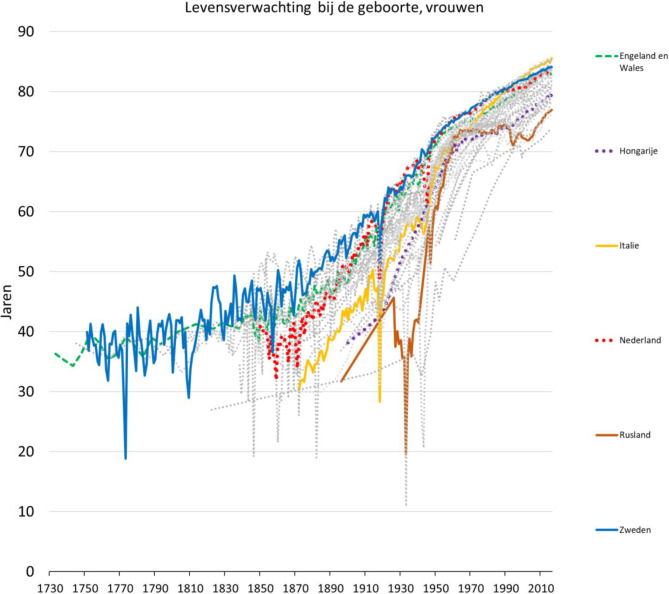

In fig. 1 zien we de ontwikkeling van de levensverwachting voor alle Europese landen waarover gegevens beschikbaar zijn. Ter vergelijking met Nederland zijn ook de trendcijfers van enkele andere landen uitgelicht. In Zweden en Engeland kan de levensverwachting al vanaf het midden van de achttiende eeuw worden gevolgd, en daar zette een geleidelijke verbetering toen al in. Ook zien we dat Nederland niet altijd tot de absolute top behoorde. Wél rond het midden van de twintigste eeuw, toen Nederland zelfs Zweden voorbijstreefde met de levensverwachting, maar niet daarvoor en ook niet meer daarna.

De toename van de levensverwachting heeft een complexe achtergrond, want hij is het resultaat van de op- en neergang van sterfte aan een groot aantal afzonderlijke gezondheidsproblemen. Zoals in het hierna volgende zal blijken, werden in een soort ‘Echternach-processie’ steeds twee stappen vooruit gedaan, wanneer de sterfte aan enkele ziekten afnam, maar werd vaak ook weer een stap terug gedaan, doordat intussen een nieuwe ziekte opkwam. In deze bijdrage zal ik dit fenomeen van opkomst en neergang van ziekten bespreken, waarbij ik ook zal ingaan op de vraag hoe het komt dat Nederland internationaal soms wel, soms niet voorop liep in het bevorderen van de volksgezondheid.

## Opkomst en neergang van ziekten

Van 43 ziekten of andere gezondheidsproblemen kan de langetermijntrend in Europa worden nagegaan, en bij niet minder dan 34 van deze ziekten is een patroon van opkomst en neergang te zien geweest. Terwijl sommige ‘opkomsten en neergangen’ zich over vele millennia uitstrekten, duurde het voor andere slechts twee decennia om zich te ontvouwen. Sommige ziekten bereikten hun hoogtepunt in de zeventiende of achttiende eeuw, andere deden dat in de negentiende of twintigste eeuw. De sterfte aan weer andere ziekten stijgt nog steeds en zal hopelijk in de toekomst gaan dalen [2].

Het feit dat zoveel ziekten een patroon van opkomst en neergang hebben vertoond, wijst op een gemeenschappelijke verklaring, en die is te vinden in de samenhang met sociaaleconomische ontwikkelingen in de brede zin van het woord. Als gevolg van een onvermoeibare drang naar verbetering van de levensstandaard wordt de mensheid voortdurend geconfronteerd met nieuwe ziekterisico’s wanneer nieuwe gebieden worden ontsloten, nieuwe productiewijzen in gebruik worden genomen of nieuwe leefgewoonten ontstaan [3, 4]. Omdat een hogere levensstandaard echter ook een groter vermogen om ziekten te beheersen met zich meebrengt, nemen deze ziekterisico’s na verloop van tijd weer af – om helaas vaak te worden vervangen door weer nieuwe ziekterisico’s. Een aantal voorbeelden kan dit illustreren.

De opkomst van infectieziekten, zoals dysenterie en andere darminfecties, begon waarschijnlijk met de komst van de landbouw in het neolithicum (rond 6.500 voor Christus in Europa), als gevolg van het feit dat mensen dichter op elkaar gingen wonen. Nadat door de verstedelijking de blootstelling aan darminfecties eerst nog veel verder was toegenomen, werden deze ziekten pas in de negentiende eeuw teruggedrongen door maatregelen zoals het aanleggen van drinkwaterleidingen en riolering, die zonder technologische vooruitgang en een toegenomen welvaartsniveau niet mogelijk waren geweest [5].

Een andere ziekte die opkwam met de landbouw is malaria, die ooit endemisch was in Europa, ook in Nederland. Pas vanaf de achttiende eeuw werd malaria teruggedrongen, als gevolg van drainage van moerassen, betere voeding, gebruik van kinine, en veel later ook van gerichte bestrijdingscampagnes, onder meer met DDT [6]. Ook een ziekte als de pest, die zich in Europa had kunnen verspreiden dankzij het toegenomen internationale handelsverkeer, was al vóór de negentiende eeuw op zijn retour, als gevolg van beschermingsmaatregelen zoals *cordons sanitaires *[7].

Door de industrialisatie en urbanisatie van Europa kwamen vervolgens weer nieuwe ziekten op, waaronder tuberculose. De geschiedenis van deze ziekte is bekend geworden door het werk van Thomas McKeown, die liet zien dat de daling van tuberculose in Engeland al lang voor de introductie van antibiotica was begonnen. Hij concludeerde daaruit dat medische zorg en ook publieke gezondheidszorg minder belangrijk zijn geweest voor de stijging van de levensverwachting, dan veel mensen in zijn tijd dachten [8]. Inmiddels weten we echter veel meer, en kunnen we de opkomst en neergang van tuberculose in meerdere Europese landen volgen. De neergang kwam waarschijnlijk niet primair door betere voeding, zoals McKeown dacht, maar vooral door menselijk ingrijpen, in de vorm van opsporing en isolatie van patiënten, betere woon- en werkomstandigheden, pasteurisatie van melk, en uiteindelijk ook antibiotica [9]. In elk geval is ook hier het vaste patroon zichtbaar, waarbij zowel de opkomst als de neergang van de ziekte direct of indirect met sociaaleconomische ontwikkeling samenhangt.

Niet alleen tuberculose, ook veel andere ziekten die met industrialisatie en urbanisatie samenhingen, raakten uiteindelijk op hun retour. Dat gold voor darminfecties als cholera en buiktyfus, voor kinderziekten als kinkhoest en difterie, voor voedingsdeficiënties als pellagra en rachitis, voor de ziekten die tot moeder- en zuigelingensterfte leiden, enzovoort [1]. In veel gevallen hebben bij die teruggang maatregelen op het gebied van de publieke gezondheidszorg een doorslaggevende rol gespeeld, maar werden die mogelijk gemaakt door algemene vooruitgang op sociaaleconomisch vlak.

**Figure 2:**
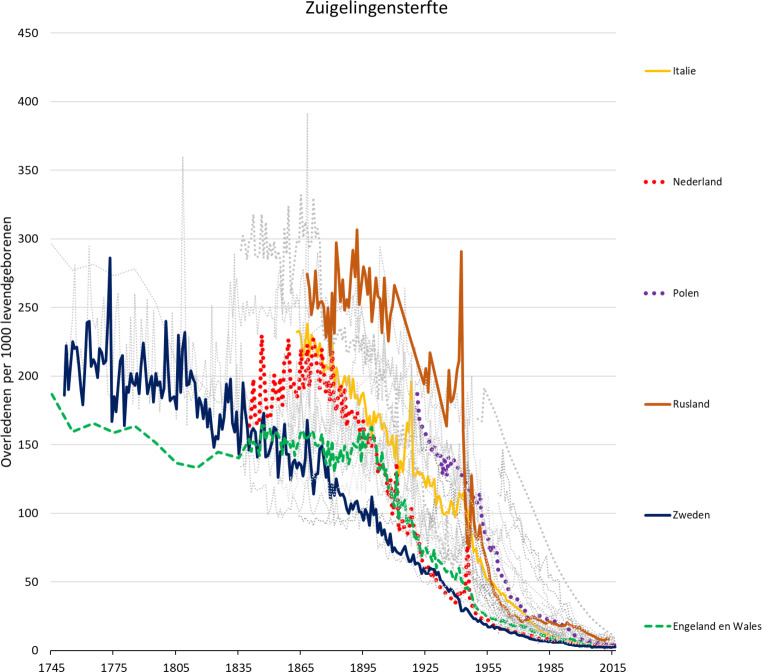

Ter illustratie is in fig. 2 de historische ontwikkeling van de zuigelingensterfte weergegeven. Net als bij de levensverwachting reiken ook hier de cijfers van Zweden en Engeland veel verder terug dan die van Nederland. De daling van de zuigelingensterfte zette in Zweden veel eerder in dan in Nederland, waar in de tweede helft van de negentiende eeuw de sterfte zelfs eerst nog beduidend toenam [10]. Maar toen de daling in Nederland eenmaal begon, was hij bijzonder steil, zodat Nederland in de jaren dertig van de twintigste eeuw Zweden (en alle andere Europese landen) voorbijstreefde. Die voorsprong ging echter na de Tweede Wereldoorlog weer verloren. Niet in de figuur te zien is dat ook het doodgeboortecijfer, dat pas in de jaren veertig sterk begon te dalen, in Nederland in de tweede helft van de twintigste eeuw aan de hoge kant bleef [11].

De ziekten van industrialisatie en urbanisatie werden vervolgens ingeruild voor weer nieuwe ziekten, die opnieuw vaak samenhingen met sociaaleconomische ontwikkelingen, in de vorm van een sterk toegenomen welvaartsniveau en de daarmee verband houdende productie- en consumptiepatronen. Na de Tweede Wereldoorlog ontstonden in heel Europa grote epidemieën van ischemische hartziekte, diabetes, diverse vormen van kanker en verkeersongevallen. Net als malaria en tuberculose zijn ook dit in zekere zin ‘ziekten van de vooruitgang’.

Kanker is hiervan een goed voorbeeld. Veel vormen van kanker zijn in de loop van de twintigste eeuw sterk toegenomen door veranderingen in leefomstandigheden en gedrag, als gevolg van wat we meestal als vooruitgang zien. Denk aan de grootschalige, industriële productie van veel wat goed voor ons is, maar ook van sigaretten, alcoholische dranken en asbest, die kanker veroorzaken. En denk aan uitstel van het krijgen van kinderen, zodat er tijd is voor het volgen van een hogere opleiding, maar ook het risico op borstkanker toeneemt [12].

**Figure 3:**
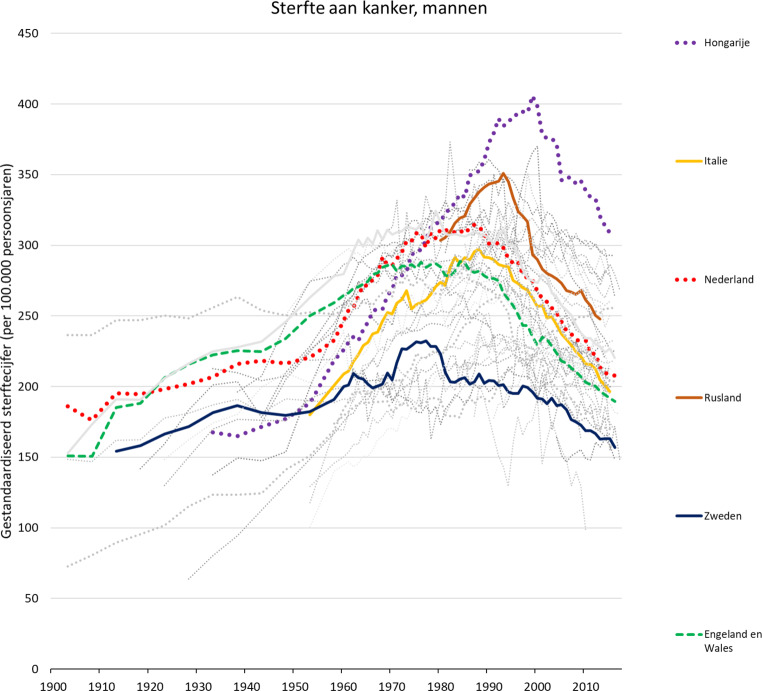

Zoals uit fig. 3 blijkt is inmiddels ook bij kanker het keerpunt gepasseerd, althans wat de sterfte betreft, mede dankzij bestrijding van sigarettenroken, vroege opsporing van bepaalde vormen van kanker en betere behandeling. Het is steeds dezelfde paradox: de wetenschappelijke vooruitgang die aan de successen in de kankerbestrijding ten grondslag ligt, was niet mogelijk geweest zonder de sociaaleconomische ontwikkeling die in een eerdere fase tot verhoogde kankerrisico’s leidde. In fig. 3 zien we dat de daling van de kankersterfte in Nederland betrekkelijk snel gaat, maar dat de piek in de kankersterfte aanzienlijk hoger was dan die in Zweden en Engeland. De belangrijkste oorzaak is sigarettenroken, dat in Nederland op zijn hoogtepunt een hogere prevalentie had en vervolgens langzamer is gedaald dan in deze andere landen [13].

Een ander belangrijk voorbeeld van een aandoening die opkwam met de naoorlogse welvaart, maar waarvan de sterfte inmiddels sterk is gedaald, is ischemische hartziekte. Net als bij kanker is de daling deels toe te schrijven aan bestrijding van risicofactoren, in dit geval roken, hoge bloeddruk en een hoog cholesterolgehalte, en deels aan verbeterde behandeling, waardoor de overleving na een hartinfarct enorm is verbeterd [14]. Diabetes en dementie zijn voorbeelden van ziekten die gedurende de laatste decennia sterk zijn toegenomen, als gevolg van respectievelijk de toename van obesitas en de uitschakeling van doodsoorzaken die vroeger op jongere leeftijd hun tol eisten, en waarbij een omslag helaas nog niet in zicht lijkt.

Hoewel sociaaleconomische ontwikkeling niet alleen aan de opkomst, maar indirect ook aan de neergang van veel aandoeningen ten grondslag ligt, speelt effectief menselijk ingrijpen bij dat laatste in veel gevallen een directe en doorslaggevende rol. In tab. 1 is een poging gedaan het relatieve belang aan te geven dat maatregelen op het terrein van de publieke gezondheidszorg, respectievelijk verbeteringen in medische zorg hebben gehad voor de neergang van de sterfte aan een groot aantal aandoeningen, op basis van wat daarover in de wetenschappelijke literatuur is gerapporteerd. Uit de tabel blijkt duidelijk dat de bijdrage van de publieke gezondheidszorg in de eerdere fasen van sterftedaling veel belangrijker was dan medische zorg. Recentelijk is de relatieve bijdrage van medische zorg echter beduidend groter geworden [15].

**Table 1:** 

		veroorzakers van dalende incidentie en/of sterfte^a^		
groep	gezondheidsprobleem	public health	medische zorg^b^	overige factoren
*gezondheidsproblemen van pre-industriële samenlevingen*	oorlog			+++++
	homicide			+++++
	honger	+		++++
	pest	++++		+
	pokken	++++		+
	tyfus	++++		+
	malaria	++	+	++
*gezondheidsproblemen van industrialiserende samenlevingen*	cholera en andere darminfecties	++++		+
	longtuberculose	++	+	++
	syfilis	++	++	+
	kinderinfecties^c^	++	+	++
	pneumonie	+	++	++
	influenza	+	++	++
	kraamvrouwenkoorts		++++	+
	zuigelingensterfte^d^	++	+	++
	doodgeboorte		++++	+
	pellagra	++	+	++
	rachitis	++	+	++
	struma	+++	+	+
	peptisch ulcus		+++	++
	appendicitis		+++	++
	pneumoconiose	++++		+
	maagkanker			+++++
*gezondheidsproblemen van welvarende samenlevingen*	ischemische hartziekte	++	+++	
	cerebrovasculaire aandoeningen	+	+++	+
	dikkedarmkanker	+	++++	
	borstkanker	++	+++	
	prostaatkanker		+++++?	
	longkanker^e^	++++		+
	levercirrose	++++		+
	verkeersongevallen	+++	+	+
	suïcide	+?	++?	++?
	aids	++	++	+

^a^ Geschatte relatieve bijdrage aan ziektespecifieke daling (som = +++++)

^b^ Inclusief preventieve interventies als onderdeel van medische zorg

^c^ Roodvonk, kinkhoest, mazelen, difterie

^d^ Specifieke oorzaken van zuigelingensterfte zijn ook inbegrepen in andere aandoeningen

^e^ Een daling is in veel landen beperkt tot mannen

## Nederland in Europese vergelijking

Wat de ontwikkeling van de levensverwachting betreft deden de meeste continentaal-Europese landen het gedurende de negentiende en twintigste eeuw aanzienlijk minder goed dan de Scandinavische landen, in het bijzonder Zweden. Zoals hierboven al werd aangegeven gold dat ook voor Nederland, althans in de tweede helft van de negentiende eeuw en in de laatste decennia van de twintigste eeuw, maar niet rond het midden van de twintigste eeuw, toen Nederland zelfs in enkele kalenderjaren de Europese recordhouder was. Wat verklaart de opmerkelijke prestatie van Nederland in de eerste helft van de twintigste eeuw, en de relatieve achteruitgang in de tweede helft?

In de negentiende eeuw was het met de volksgezondheid in Nederland niet goed gesteld. West-Nederland kende toen een zeer hoge sterfte, wat overigens al in de achttiende eeuw bekend was. Volgens de Britse demograaf Thomas Malthus (1766–1834) was Nederland zelfs ‘het graf van Duitsland’, omdat zoveel Duitse immigranten er hun vroegtijdige dood vonden. Deze erbarmelijke situatie stond in schril contrast met het roemrijke verleden van Nederland: in de zeventiende eeuw was de Republiek der Zeven Verenigde Nederlanden het meest welvarende, verlichte en machtige land van Europa. Religieus geïnspireerde scholingscampagnes tijdens en na de Tachtigjarige Oorlog (1568–1648) hadden ook het geletterdheidsniveau naar recordhoogten gebracht. In het begin van de achttiende eeuw verloor de Nederlandse Republiek haar dominante positie in de wereldhandel echter aan Groot-Brittannië, en de daaruit voortvloeiende achteruitgang van de economie werd nog verergerd door de schade die werd aangericht door de Napoleontische oorlogen in het begin van de negentiende eeuw [16].

Meer specifiek was de hoge sterfte in Nederland in de negentiende eeuw waarschijnlijk te wijten aan een combinatie van hoge bevolkingsdichtheid en gebrek aan goed drinkwater, die beide het risico op infectieziekten vergrootten. Verder kwamen maatregelen op het gebied van de publieke gezondheid in Nederland traag op gang. Deze situatie veranderde pas rond 1870, toen als eerste teken van vooruitgang op het terrein van de volksgezondheid de kindersterfte begon af te nemen, waarschijnlijk meer als gevolg van culturele veranderingen (zoals de verspreiding van moderne concepten van hygiëne en kinderverzorging), dan als gevolg van economische groei. Een hoog niveau van geletterdheid – een overblijfsel van het glorieuze verleden van Nederland – zal deze snelle verbeteringen mede mogelijk hebben gemaakt [17].

In de eerste helft van de twintigste eeuw werden trends in de levensverwachting nog sterk bepaald door trends in zuigelingensterfte. Door een opmerkelijke daling van de zuigelingensterfte tot internationaal gezien zeer lage niveaus (zie fig. 2) steeg de Nederlandse levensverwachting tot recordhoogte. Deze gunstige situatie hield aan tot het begin van de jaren zestig, en omdat dit ook in de rest van de wereld niet onopgemerkt bleef, werd door de Amerikaanse overheid aan Nederlandse volksgezondheidsexperts gevraagd om dit opmerkelijke succes te verklaren. Zij concludeerden dat de gunstige situatie in Nederland een afspiegeling was van een lange traditie van goede kinderzorg in het gezin, geholpen door wijkverpleegkundigen en consultatiebureaus, en ondersteund door sociale zekerheidsmaatregelen en een stijgende levensstandaard [18]. Andere analyses hebben daarnaast gewezen op het feit dat Nederland eerder dan veel andere Europese landen een landelijk systeem van goed opgeleide verloskundigen had ingevoerd [19].

Dat Nederland – in het derde kwart van de negentiende eeuw nog achterlopend op meer geavanceerde Europese landen – erin slaagde om zich bij de Scandinavische landen aan de top van de Europese ranglijst te voegen, zal deels verklaard worden door de snelle groei van de Nederlandse economie, zowel voor, tijdens als direct na de Eerste Wereldoorlog. Dit kwam door een combinatie van factoren, waaronder het feit dat Nederland profiteerde van snelle economische groei in het directe achterland, namelijk de belangrijkste handelspartner Duitsland [20]. Een snelle economische ontwikkeling is echter zeker niet de enige verklaring, want in de jaren twintig van de vorige eeuw waren de zuigelingensterfte en de levensverwachting in Nederland veel gunstiger dan op basis van het welvaartsniveau kan worden verklaard. Anders dan in Zweden speelde de staat slechts een bescheiden rol bij de verbetering van de volksgezondheid in Nederland, en berustte het initiatief veelal bij het ‘maatschappelijke middenveld’ [21]. Het was de ‘verzuiling’ van de Nederlandse samenleving, dat wil zeggen de organisatie van het sociale leven in afzonderlijke protestantse, rooms-katholieke en socialistische ‘zuilen’, die een succesvol ‘beschavingsoffensief’ binnen elke zuil stimuleerde dat alle aspecten van het leven omvatte, inclusief de zorg voor baby’s en kinderen. ‘Particulier initiatief’ creëerde ook binnen elke ‘zuil’ aparte preventieve en curatieve zorgorganisaties.

Helaas verloor Nederland zijn voorsprong in de jaren tachtig en negentig van de twintigste eeuw, toen in sommige leeftijdsgroepen in Nederland een volledige stagnatie van de sterftedaling optrad, terwijl veel andere Europese landen hun snelle sterftedaling voortzetten. Deze stagnatie was zowel bij de allerjongsten (perinatale sterfte, dus doodgeboorte plus eersteweeksterfte), als bij de alleroudsten (sterfte onder 80-plussers) te zien.

De verloskundige beroepsgroepen schreven aanvankelijk de hoge perinatale sterfte toe aan een meer volledige registratie in Nederland, maar dit standpunt werd onhoudbaar met de publicatie van internationaal geharmoniseerde cijfers, die bevestigden dat Nederland ongunstig afstak bij andere Europese landen [22]. Meer gedetailleerde onderzoeken toonden aan dat stagnatie van de daling van de perinatale sterfte het gevolg was van een gebrek aan vooruitgang in de perinatale zorg [23]. Dit kwam doordat een systeem dat in belangrijke mate afhankelijk was van verloskundigen, minder goed in staat was om nieuwe hightechdiagnostiek en interventies te implementeren. Het voordeel dat Nederland in een eerdere fase van sterftedaling had genoten, in de vorm van uitstekende eerstelijnsverloskunde, was in een latere fase dus omgeslagen in een nadeel. Toen de regering er eindelijk van overtuigd raakte dat er iets moest gebeuren, werd er een regeringscommissie geïnstalleerd die een betere integratie van eerstelijns- en tweedelijnsverloskundige zorg adviseerde.

De stagnatie van de sterftedaling onder ouderen was deels het gevolg van een gebrek aan vooruitgang bij het terugdringen van roken, dat extreem hoge niveaus had bereikt onder de Nederlandse geboortecohorten die in de jaren tachtig en negentig overleden (zie hierboven). Een vergelijkbare stagnatie van de sterftedaling onder ouderen deed zich voor in Denemarken, ook deels als gevolg van een historisch hoge prevalentie van roken [24, 25]. Een intrigerende overeenkomst tussen Nederland en Denemarken is dat beide landen kleine handelsnaties zijn, waarin zich mede onder invloed van de eisen van de handel een libertaire traditie heeft ontwikkeld, die collectief ingrijpen in het rookgedrag van de burger in de weg staat. Deze libertaire trek heeft in Nederland bijgedragen aan de totstandkoming van rationeel beleid op controversiële gebieden als heroïneverslaving, aidspreventie en euthanasie, maar heeft zoals uit dit voorbeeld blijkt ook nadelen.

Behalve een tekortschietend antirookbeleid hebben vermoedelijk ook budgettaire beperkingen in de gezondheidszorg een rol gespeeld bij de stagnatie van de sterftedaling onder ouderen in Nederland. Deze budgettaire beperkingen leidden in de jaren tachtig en negentig tot een minder snelle groei van de zorguitgaven in Nederland dan in andere Europese landen, maar remden ook de introductie van nieuwe effectieve behandelingen af. Na 2002, toen de regering had besloten de budgettaire beperkingen op te heffen, begon zowel het gebruik (en de kosten) van de gezondheidszorg als de levensverwachting weer snel te stijgen [26].

## Beschouwing

Voor de publieke gezondheidszorg in Nederland heeft deze geschiedenis zowel bevredigende als teleurstellende kanten. Het is, om met het goede nieuws te beginnen, goed om nog eens vast te stellen dat de publieke gezondheidszorg een cruciale rol heeft gespeeld bij de toename van de levensverwachting bij de geboorte, niet alleen in het verre verleden, maar ook in de laatste decennia van de twintigste eeuw. Het ging hierbij overigens om publieke gezondheidszorg in de ruime zin van het woord, dus niet beperkt tot instellingen met publieke gezondheidszorg in hun missie, en al helemaal niet tot professionals met een opleiding op dit terrein. De aanleg van drinkwaterleidingen en riolering, maatregelen op het gebied van de verkeersveiligheid, en het heffen van accijnzen op alcohol en tabak vallen immers geheel of grotendeels binnen het domein van andere instellingen of disciplines.

Ook het feit dat de publieke gezondheidszorg de verantwoordelijkheid voor successen in de volksgezondheid tegenwoordig moet delen met de medische zorg moet tot enige bescheidenheid manen. In tegenstelling tot wat velen op gezag van McKeown nog steeds aannemen, is medische zorg met al zijn technisch vernuft, in combinatie met een verzekeringsstelsel dat een goede financiële toegankelijkheid regelt, uitgegroeid tot een effectief instrument om de gezondheid van de hele bevolking te bevorderen. De spectaculaire daling van de sterfte aan ischemische hartziekte sinds de jaren zeventig van de twintigste eeuw was zonder de cardiologie niet mogelijk geweest. Voorkómen is nog steeds beter dan genezen, en vaak ook goedkoper, maar helaas is preventie lang niet altijd mogelijk, en is er ook niet altijd voldoende draagvlak voor preventief beleid, en dan is het goed dat er ook curatieve mogelijkheden zijn.

Voor de publieke gezondheidszorg in Nederland waren, achteraf gezien, de decennia rond het midden van de twintigste eeuw de ‘gouden jaren’, althans wanneer we het succes van deze sector afmeten aan uitkomstmaten als zuigelingensterfte en levensverwachting. Op een of andere manier is het echter niet gelukt dit succes goed vast te houden, dat wil zeggen een succesformule te vinden die past bij de gezondheidsproblemen van de huidige tijd. Dat blijkt ook uit een recent onderzoek waarin werd nagegaan hoe de resultaten van het Nederlandse gezondheidsbeleid zich in de periode 1970–2010 verhielden tot die van andere Europese landen. Hierbij werden de nationale prestaties vergeleken op tien verschillende terreinen van preventief gezondheidsbeleid, namelijk roken, alcohol, voeding, zwangerschap en bevalling, jeugdgezondheidszorg, infectieziekten, hypertensie, kankerscreening, verkeersveiligheid en luchtverontreiniging. In de Europese rangorde op basis van een samenvattende score stond Nederland op de vijfde plaats, na Zweden, Noorwegen, IJsland en Finland [27]. De conclusie was dat ‘Nederland een van de succesvolste Europese landen [is] wat preventief gezondheidsbeleid betreft, [maar dat] om op alle terreinen uit te blinken […] meer inspanningen nodig [zijn]’ [28].

De kans lijkt mij groot dat internationaal-vergelijkende onderzoeken van de uitkomsten van het beleid gevoerd tijdens de COVID-19-pandemie tot vergelijkbare conclusies zullen leiden. Over de jaren 2020 en 2021 lag de aan COVID-19 toe te schrijven ‘oversterfte’ in Nederland op het (West‑)Europese gemiddelde, en beduidend boven die in de Scandinavische landen [29]. De publieke gezondheidszorg Nederland moet beter kunnen, maar hoe? Een eenvoudig antwoord op die vraag is er niet, omdat zoals hierboven aangeduid ook culturele belemmeringen een rol spelen. Maar meer landelijke aansturing, een stevigere wettelijke basis en ruimere financiering zullen zeker helpen [30].
